# A Review on Friction Stir Welding of High-Strength Al-Zn-Mg Alloy: Insights on Second-Phase Particles

**DOI:** 10.3390/ma17205107

**Published:** 2024-10-19

**Authors:** Keqi Wang, Anton Naumov, Evgenii Panchenko, Oleg Panchenko

**Affiliations:** Laboratory of Lightweight Materials and Structures, Institute of Machinery, Materials, and Transport, Peter the Great St. Petersburg Polytechnic University, St. Petersburg 195251, Russia; anton.naumov@spbstu.ru (A.N.); panchenko.ev@edu.spbstu.ru (E.P.); panchenko_ov@spbstu.ru (O.P.)

**Keywords:** Al-Zn-Mg alloy, intermetallic constituent particles, dispersoids, strengthening precipitates, friction stir welding, microstructure evolution

## Abstract

The friction stir welding (FSW) process is a unique combination of deformation and high temperature, which provides opportunities to modify microstructures through the adjustment of the processing parameters and is an ideal way to join non-weldable aluminum alloys by avoiding the formation of a molten pool. The 7xxx series heat-treatable aluminum alloys are widely used in the aerospace field as high-performance structural materials. The microstructure evolution and mechanical performance of these alloys are affected by the effects of thermomechanical processing, which provides opportunities to optimize the material properties by controlling microstructural features such as intermetallic constituent particles, dispersoids and nanoscale precipitates. This paper focuses on the basic principles of the thermal and mechanical effects generated during FSW on the evolution of second-phase particles in different zones of the weld.

## 1. Introduction

Heat-treatable Al-Zn-Mg alloys are widely used in the aerospace industry due to the high strength, low density, high crack resistance and good thermal stability of their microstructures. Since components must withstand a variety of challenging loading conditions, the demand for joining different materials in various industries is growing. However, various defects such as cracks, voids, pores, and inclusions appear in the traditional fusion welding process for Al-Zn-Mg-Cu alloys, which seriously affect the mechanical properties of the joints [1,2].

The friction stir welding (FSW) process is a widely used and inventive solid-state joining technique that does not form a melt pool, making it a method well-suited for joining low-melting-point alloys such as aluminum. During FSW, materials are softened and joined together at temperatures below the corresponding melting temperatures of the materials, resulting in higher mechanical properties of the weld zone compared to those obtained when using fusion welding, especially when the focus is on heat-treatable lightweight alloys. Undesirable microstructures due to melting and resolidification are eliminated in friction stir welds, resulting in improved mechanical properties, including ductility and strength. Thus, these welds are characterized by lower residual stresses, the absence of microdefects and high dimensional stability [3,4,5]. Compared with fusion welds, the tensile strength of friction-stir-welded joints increases by about 15–20%, the ductility almost doubles and the fracture toughness increases by more than 30% [6,7]. FSW effectively solves the problem of finding a welding method for lightweight materials, expanding the range of material choices in the design process.

Thermal processes, such as the heating rates and cooling rates, as well as mechanical processes, such as plastic deformation and material flow, affect the microstructure of the weld. Due to the thermal cycle that occurs during welding, the strengthening precipitates in the material may become coarser or dissolve, resulting in a decrease in joint strength and the formation of softening zones in the weld [8,9,10,11]. Therefore, it is necessary to study the precipitation behavior of the strengthening phases in welds.

## 2. Al-Zn-Mg Heat-Treatable Alloy

**Figure 1:**
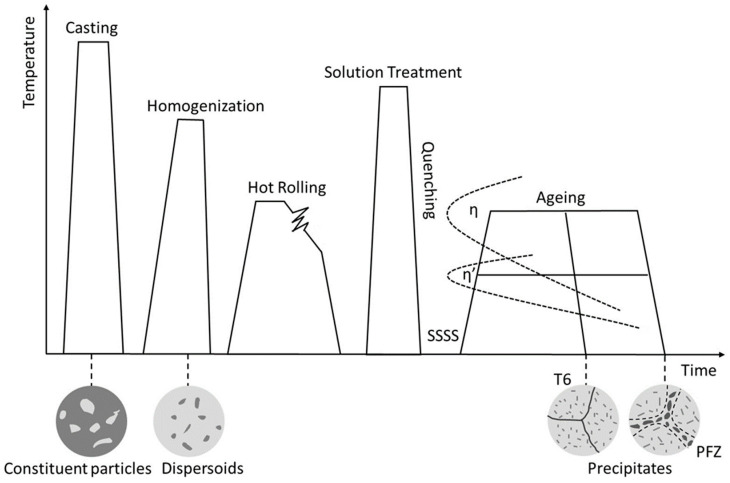
Schematic processing route of Al-Zn-Mg heat-treatable aluminum alloy sheets.

Alloying elements in aluminum are either in a solid solution state or segregated as second-phase particles and are usually divided into three categories according to the function they perform and the temperature range of their formation: constituent particles, dispersoids and precipitates. Their sizes are usually in the ranges of 0.5–50 µm, 0.1–1 µm and 0.005–0.1 µm, respectively [12,13]. Alloy properties can be controlled through proper alloying and processing. The processing stages for heat-treatable Al-Zn-Mg-series aluminum alloy sheets are shown in Figure 1.

### 2.1. Intermetallic Constituent Particles in Al-Zn-Mg Alloys

Non-equilibrium solidification during aluminum alloy casting causes micro- and macro-segregation. Micro-segregation can be removed during homogenization, while macro-segregation cannot be redistributed by subsequent heat treatment. During the solidification process in aluminum ingots, the primary phase formed by the liquid–solid eutectic reaction between the arms of α-Al dendrites is called “constituent particles” [14]. Their size, quantity, distribution and composition depend on the solidification rate, the dendrite size and the contents of alloying elements and impurity elements, which can be transformed during further high-temperature heat treatment, such as homogenization or solution heat treatment. They are large, irregular in shape and very diverse in chemical composition, containing different amounts of Al, Cu, Fe, Mn and Si and characteristically crystallizing into different atomic structures [15].

Depending on whether the constituent particles are soluble in the subsequent heat treatment process, they can be divided into two categories: insoluble constituent particles and soluble/partially soluble constituent particles. Insoluble constituent particles are usually rich in Fe, since Fe is difficult to remove from molten metal and is poorly soluble in Al [16,17]. Large insoluble constituent particles have a negligible effect on strength (the incoherent ratio of constituent particles to the matrix and their larger size virtually eliminate strengthening) but have a negative impact on ductility and fracture toughness since the particles are brittle and easily fragment or may be broken during processing [18]. During the deformation process, broken intermetallic particles may serve as initiation points for microcracks, which have an extremely negative effect on the fracture toughness of alloys [19]. The constituent particle size can be reduced by increasing the solidification rate, reducing the Fe and/or Si content (inevitable impurities in Al alloys) or increasing the degree of deformation during mechanical and thermomechanical processing [12,20].

It is common for small amounts of Fe to be present as an impurity in commercial Al alloys. In the case of low Fe impurity levels, most of the Fe remains in solid solution, and when the eutectic reaction occurs, α-Al and Al_3_Fe intermetallic compound particles with a monoclinic crystal structure are produced. For Al-Zn-Mg alloys with low Si contents, Fe exists in the form of Al_3_Fe, while at higher Si contents, α-AlFeSi is formed first. Al_7_Cu_2_Fe is formed in 7xxx alloys with high Cu content. High-Si-content phases (such as Mg_2_Si and SiO_2_) and high-Mn phases (such as Al_6_ (Fe, Mn), Al_5_Si_2_ (Fe, Mn) and Al_3_ (Fe, Mn, Cr)) can be also formed in Al-Zn-Mg alloys [21]. Although Si has noticeable solubility in Al, its solubility is reduced by alloying elements, especially Mg; therefore, although Mg_2_Si is soluble in some alloys, it is practically insoluble in Al-Zn-Mg alloys [22].

For soluble/partially soluble constituent particles with high concentrations of alloying elements, solid-solution processing for a longer time or at a higher temperature can reduce the number of particles. The Cu content is usually controlled in 7xxx aluminum alloys to limit the formation of large intermetallic particles such as the S-phase (Al_2_CuMg) and θ-phase (Al_2_Cu) [23,24].

### 2.2. Dispersoids in Al-Zn-Mg Alloys

Dispersoid particles are formed during homogenization. Since dispersoids are formed by solid–solid reactions, they are coherent with at least one interface of the matrix. The tendency towards recrystallization is counteracted by Zener pinning, which is created by dispersoids at migrating boundaries [25]. In aluminum alloys used for structural applications, their primary role is to suppress recrystallization and grain growth, controlling the grain size during high-temperature heat treatment and thermomechanical processing.

**Figure 2:**
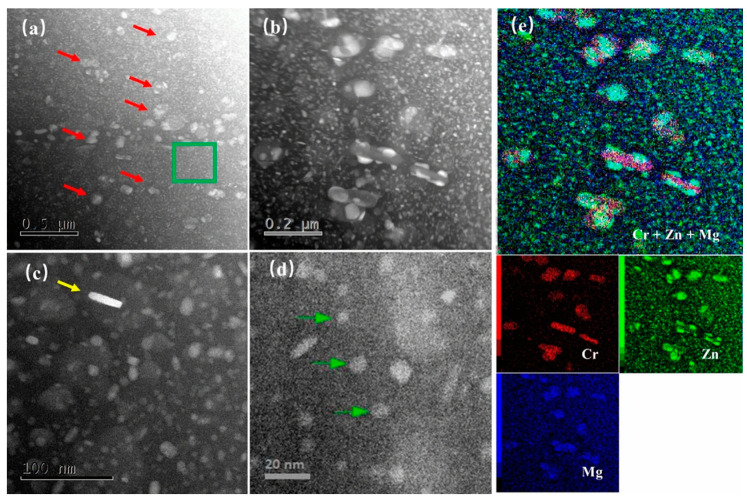
HAADF-STEM images of dispersoids and precipitates in single-peak aged 7075 aluminum alloy: (**a**) precipitates (green box) and dispersoids (red arrows); (**b**) MgCu_x_Zn_2−x_ nucleated on Al_18_Mg_3_Cr_2_ dispersoids; (**c**) an η precipitate (yellow arrow) among a high density of fine η′ precipitates; (**d**) a large quantity of fine η′ precipitates (green arrows); (**e**) elemental mapping (EDS) of (**b**) [28].

The dispersoids can not only suppress recrystallization and grain growth to improve the fracture toughness of alloys but also serve as nucleation centers for strengthening precipitates [26,27]. As shown in Figure 2, the MgCu_x_Zn_2−x_ phases are attached to the Al_18_Mg_3_Cr_2_ (E-phases) dispersoids in 7075 aluminum alloy [28]. Scanning electron microscopes (SEMs) using backscattered electrons (BSEs) and transmission electron microscopes (TEMs) are commonly used to determine the size distribution of nanoscale phases such as dispersoids. Dispersions appear brighter than the main substance in grayscale images [29].

In 7xxx aluminum alloys, transition elements such as Cr, Mn and Zr form dispersoids during the homogenization process. The volume fraction of the dispersoids is approximately 0.05–0.2%, and the typical particle size range is approximately 20~500 nm. In Cr-containing alloys, dispersoids such as Al_18_Mg_3_Cr_2_ (E-phase) or Al_12_Mg_2_Cr with a size of 50~300 nm can be formed to prevent the nucleation and grain growth of recrystallized grains in the aluminum alloy during processing [23,30]. A trace amount of Cr (0.1–0.2%) is beneficial to improving the stress corrosion resistance of aluminum alloys and combines well with Cu [31]. Al_3_Zr phases (size: 20~60 nm) are present in Zr-added alloys [32,33], while Al_6_Mn and Mn_3_Si_2_Al_15_ are present in Mn-containing alloys. The Al_6_Mn particles generated in 7xxx aluminum alloys can improve the alloys’ stress corrosion resistance [34]. Cr- or Mn-based dispersoids are generally incoherent with the matrix, whereas Zr-containing dispersoids are coherent with the matrix. The fracture toughness of the alloy increases with a change in the dispersoid type from Mn-based to Zr-based; this may be due to a decrease in the average dispersoid size. The actual effect of the dispersoid type on fracture toughness is quite complex, taking into account other factors such as an increase in the dispersoid phase modulus and the differences in dispersoid particles [35].

### 2.3. Precipitates in Al-Zn-Mg Alloys

Precipitates are nanoscale fine phases or clusters that form during aging. Factors such as the composition, coherence, volume fraction, size and distribution of precipitates determine their effect, and their properties depend on the type and concentration of alloying elements, as well as the heat treatment conditions.

Three types of matrix precipitates are formed during aging heat treatment: Guinier–Preston zones (GP zones), thermal-sensitive precipitates and equilibrium precipitates.

The initial GP zones are considered to be coherent with the matrix. During heat treatment, the precipitates undergo a transformation, increasing in size and average spacing, which causes them to gradually lose their coherence and become semi-coherent η′ phases and incoherent η phases in 7xxx aluminum alloys. The improvement in alloy strength is related to an increase in the proportion of small-scale precipitates, reaching an optimal distribution at maximum hardness. This is followed by a decrease in hardness associated with the coarsening of the precipitates, which leads to a gradual decrease in the number of particles and an increase in the average solubility between particles [35]. The yield strength of high-strength 7xxx aluminum alloys is typically in the range of 450–570 MPa.

Aging is the main means to optimize the microstructure and comprehensive properties of 7xxx aluminum alloys. After solution treatment and quenching, the alloy elements remain in a supersaturated state, and the dislocation density of the alloy is relatively high. During the cooling process at room temperature, the fine GP zone in a 7xxx aluminum alloy is easily separated from the alloy, thereby strengthening it.

Peak aging is an effective way to achieve maximum alloy strength by isolating fine and dispersed strengthening phases inside the grains to prevent the movement of dislocations during deformation. In 7xxx aluminum alloys, both the GP zone and η′ phase can play a strengthening role [36]. At an aging temperature lower than the dissolution point of the GP zone, by controlling the heating rate, a large amount of the GP zone is preferentially precipitated in the grains and then transformed into η′ strengthening phases.

However, the continuous chain-like grain boundary phases present in the T6-state Al-Zn-Mg alloy can easily serve as continuous channels for corrosion expansion, increasing the local corrosion sensitivity of the alloy [36]. Through double-stage aging (over-aging), at the expense of some alloy strength, a discontinuously distributed coarse phase is formed at the grain boundary to improve the corrosion resistance of 7xxx aluminum alloys. In order to produce aluminum alloys with good corrosion resistance, without losing too much strength, a three-step aging (pre-aging → retrogression → re-aging) method was proposed that not only ensures the distribution of fine precipitates inside the grains but also forms discontinuous phases at the grain boundaries. Note that over-aging and three-step aging produce a particle-free zone (PFZ) at the grain boundaries.

The formation sequence for 7xxx aluminum alloy precipitates is generally accepted to be as follows [19,30,37,38,39,40,41,42]:

Supersaturated solid solution (SSSS) → GP zone→ η′ → η/T (Al, Zn)_49_Mg_32_ (in alloys with a relatively high Mg content).

## 3. Al-Zn-Mg Alloy Joined by Friction Stir Welding

**Figure 3:**
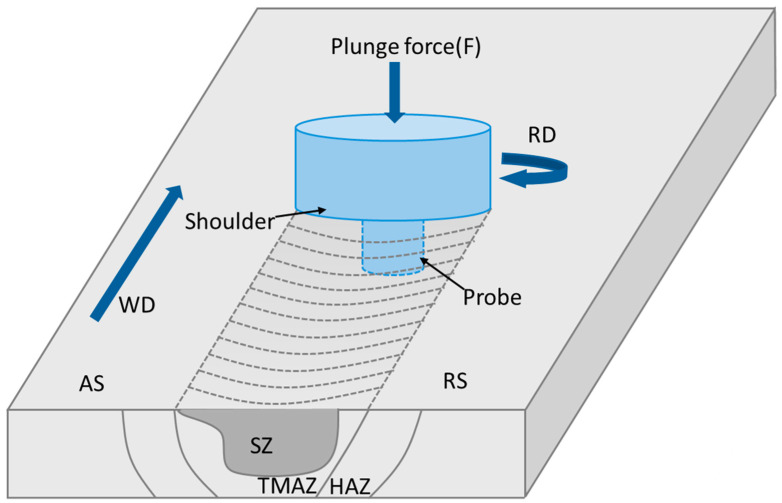
Principle of FSW process and weld zones.

FSW is a technique for joining materials in solid state by using non-consumable tools. The tool used in the process consists of a shoulder and a probe, such that the shoulder contacts the workpiece. Heat, created by friction, causes the material to soften and flow in reverse compared to the motion of the tool. Due to the action of the shoulder and probe of the rotating tool, mechanical processes such as plastic deformation, material flow and thermal cycles occur to varying degrees in different zones of the weld. The cross-section of the friction stir weld (FSWeld) consists of the base metal (BM), heat-affected zone (HAZ), thermo-mechanically affected zone (TMAZ) and stir zone (SZ) (Figure 3). Based on the characteristics of heat-strengthened aluminum alloys, it is necessary to study the precipitation transformation in the weld to understand the influence of precipitate evolution on the mechanical properties of the weld.

Due to the heat input associated with FSW in heat-treatable aluminum alloys, metallurgical phenomena may occur in individual joint areas, such as aging, over-aging, precipitate growth or dissolution.

The microstructural changes in friction-stir-welded joints are always accompanied by softening. It is well known that the mechanical properties of heat-treatable Al-Zn-Mg alloys depend mostly on the η′ metastable precipitates. Therefore, changes in the hardness of the entire joint are closely related to the type, shape and distribution of the precipitates.

### 3.1. Evolution of Constituent Particles in Al-Zn-Mg Alloys During FSW

**Figure 4:**
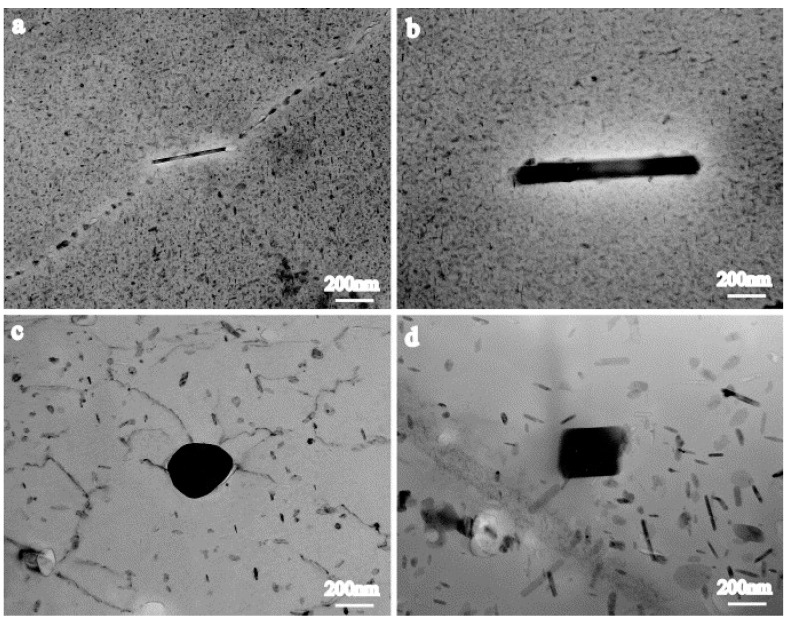
Morphologies of Al_7_Cu_2_Fe in 7050-T651 base metal (**a**,**b**) and in stir zone of FSWeld (**c**,**d**) [43].

The insoluble constituent particle Al_7_Cu_2_Fe was detected and confirmed using a TEM in the 7050-T651 alloy. The large rod-shaped Al_7_Cu_2_Fe particle from the base metal was crushed during FSW and then coarsened into spherical or blocky shapes under the influence of the thermomechanical cycle process, as shown in Figure 4 [43]. In studies of the FSW of other aluminum alloy series, it was also mentioned that due to the severe plastic deformation caused by the stirring action of the tool, larger insoluble intermetallic constituent particles were crushed into smaller particles [44,45,46].

Soluble constituent particles dissolve in the SZ and TMAZ after undergoing thermocycling during the FSW process, and their quantity decreases, which has a beneficial effect on the toughness. Thermally stable constituent particles are crushed and redistributed in the weld under the stirring action of the tool during FSW, so, in principle, their volume fraction should be maintained. However, some researchers [45] have noticed that the volume fraction of thermally stable constituent particles in aluminum alloys decreases; a more likely explanation is that the insoluble constituent particles are crushed or eroded, such that the particle size is less than the detection limit. The particle erosion is manifested by the more rounded shape of the particles in the SZ. Fragmentation of the brittle constituent particles occurs during FSW; these brittle fragments will preferentially form cracks during the service life of the materials, reducing fracture toughness of the weld.

### 3.2. Evolution of Dispersoids in Al-Zn-Mg Alloys During FSW

**Figure 5:**
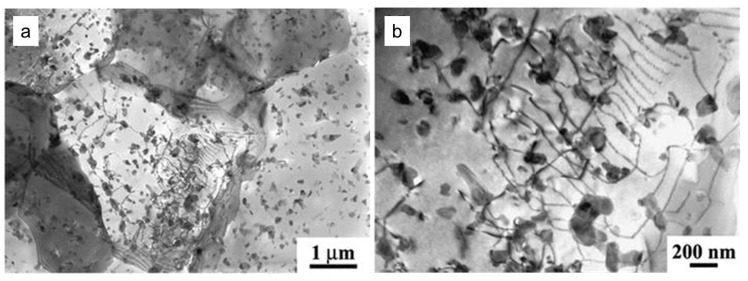
TEM images of SZ-FSW 7075Al-T651 join: (**a**) randomly distributed dispersoids (Mg_3_Cr_2_Al_18_); (**b**) dispersoids pinning dislocations [48].

Dispersoids containing refractory elements are thermally stable during subsequent heat treatment. Al_18_Mg_3_Cr dispersoids in 7075-T6 aluminum alloy were observed in the dynamic recrystallization zone (stir zone) of a weld, which experiences extremely high temperature and severe plastic deformation, after high-speed FSW [47]. A large amount of Mg_3_Cr_2_Al_18_ dispersoid was randomly distributed and pinned dislocations in the SZ of a 7075-T651 FSWeld (Figure 5) [48]. A TEM investigation of the stir zone after FSW of 7050-T451 alloy showed a high dislocation density in some grains, and they were pinned by Al_3_Zr dispersoids [46].

**Figure 6:**
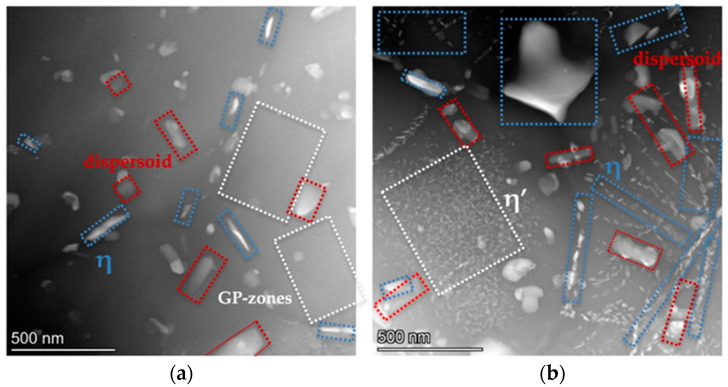
Precipitation behavior of 7075-T6 in SZ of FSWeld (**a**) and IFSWeld (**b**) [49].

In a study of impulse friction stir welding (IFSW) of 7075-T6 aluminum alloy, the Cr- and Mn-containing dispersoids from the base material were not affected by the thermal cycle during the FSW process. Some dispersoids were covered with η phase, which provided evidence that dispersoids served as the nucleation sites for the precipitates (Figure 6) [49].

According to several studies on the FSW of heat-treatable aluminum alloys [29,43,50,51,52,53,54,55,56,57], the dispersoids are thermally stable and basically will not dissolve andwill not be crushed by the thermomechanical action of FSW. The dispersoids are homogenously distributed in the weld and have no direct strengthening effect on the weld, but they can serve as nucleation sites for strengthening precipitates and promote dispersion strengthening in aluminum alloys.

### 3.3. Evolution of Precipitates in Al-Zn-Mg Alloys During FSW

The temperature gradient of FSW causes a complex range of precipitate formation from the weld center of dynamic recrystallization to the unaffected base metal. Due to the thermal sensitivity of these precipitates, different microstructures form in different zones of the weld, which leads to different strengthening behaviors and a non-uniform hardness distribution in the weld. Various studies have reported that the peak temperatures in different zones range from approximately 200 to 480 °C [58,59,60].

GP zones provide moderate hardening, and the heat-sensitive η′ phases are transformed from the GP zones, typically found in peak aged alloys and associated with better mechanical properties of the alloys [36]. Stable η precipitates (MgZn_2_) correspond to the over-aged state and are related to a reduction in the mechanical properties of the alloy. Due to the different thermal and mechanical conditions in different zones of the FSWeld, the precipitation behavior of heat-treatable aluminum alloys varies, and the hardness distribution of the weld usually presents a W shape [8,9,10,11,47].

#### 3.3.1. Stir Zone (SZ)

The GP zones form at low temperatures (20–125 °C) and dissolve at 100–125 °C. The transformation of the GP zones into the η′ phases is the main mechanism for the formation of η′ precipitates, and the nucleation of η′ precipitates can also occur directly at higher temperatures (from ~130 to 290 °C) [59]. The SZ of a 7075-T651 alloy friction-stir-welded joint experienced the highest heat input, with a peak value likely exceeding 480 °C [58], and severe plastic deformation; thus, a strong precipitate strengthening-phase evolution occurred in the SZ. It is reported that the dissolution temperature range of the strengthening phases in 7050-T7451 is lower than the peak temperature produced by FSW, which allows the strengthening phases to be completely dissolved in the SZ of the joint [46].

The reason for the absence of strengthening precipitates during the post-weld cooling process is that the cooling rate is too high, so the dissolved precipitates do not have enough time to reprecipitate and remain in α-Al in a supersaturated solid solution state [43,60,61,62,63,64,65,66,67,68]. The supersaturated strengthening phases dissolved in the aluminum matrix can only be reprecipitated through appropriate post-weld heat treatment (PWHT) [11,57,69,70,71]. An AA7075-T6 alloy friction-stir-welded joint was subjected to cyclic heating in the range of 400–480 °C for 1.5 h, and then artificial aging was performed at 130 °C for up to 36 h. This PWHT dissolved the coarse η precipitates in the weld, promoted the precipitation of a fine strengthening phase and improved the mechanical properties of the joint [70]. The completely dissolved strengthening precipitates in the SZ of the AA7075-T651 friction-stir-welded joint after solution treatment and artificial aging (480 °C, 1 h + 120 °C, 24 h) were reprecipitated, improving the tensile properties and hardness of the joint [71].

The microstructural changes in an AA7075 FSWeld were assessed by Feng A. H. et al. [53]. FSW led to significant grain refinement and the dissolution of η′ phases (Mg (Zn, Al, Cu)_2_) in the SZ, while the dispersoids (E-phases) remained virtually unchanged. The precipitate evolution during FSW of Al-Zn-Mg-Cu alloy was studied through in situ measurements coupled with modeling by J.F. dos Santos et al. [72]. A large number of evenly distributed, fine η′ precipitates with a diameter of several nanometers were observed in the original microstructure of the AA7449 alloy which disappeared in the weld. SZ. Zhao, Y. et al. performed repeated friction stir spot welding of Al-Zn-Mg-Cu alloy [73]; in the SZ, GP zones and η′ precipitates were completely dissolved in the aluminum matrix under the influence of the welding heat, while stable η precipitates and dispersoids remained. The hardness reduction in the SZ is also due to the dissolution of GP zones and η′ strengthening precipitates. The above studies agreed that the GP zones and heat-sensitive η′ strengthening phases of the base material after FSW are completely dissolved in the SZ. The dissolution of the strengthening phases reduces the hardness of the SZ; note that this reduction in hardness is partially offset by the formation of a fine grain structure in the SZ. The hardness evolution of the SZ domain is usually plateau-like [74].

Alexander Kalinenko [75], on the other hand, indicated that FSW of aluminum alloys has a wide peak welding temperature range of 150~450 °C. Part of this temperature range lies below the particle dissolution temperature. In low-temperature welds, strengthening precipitates may not dissolve or may only partially dissolve.

#### 3.3.2. Thermomechanically Affected Zone (TMAZ)

**Figure 7:**
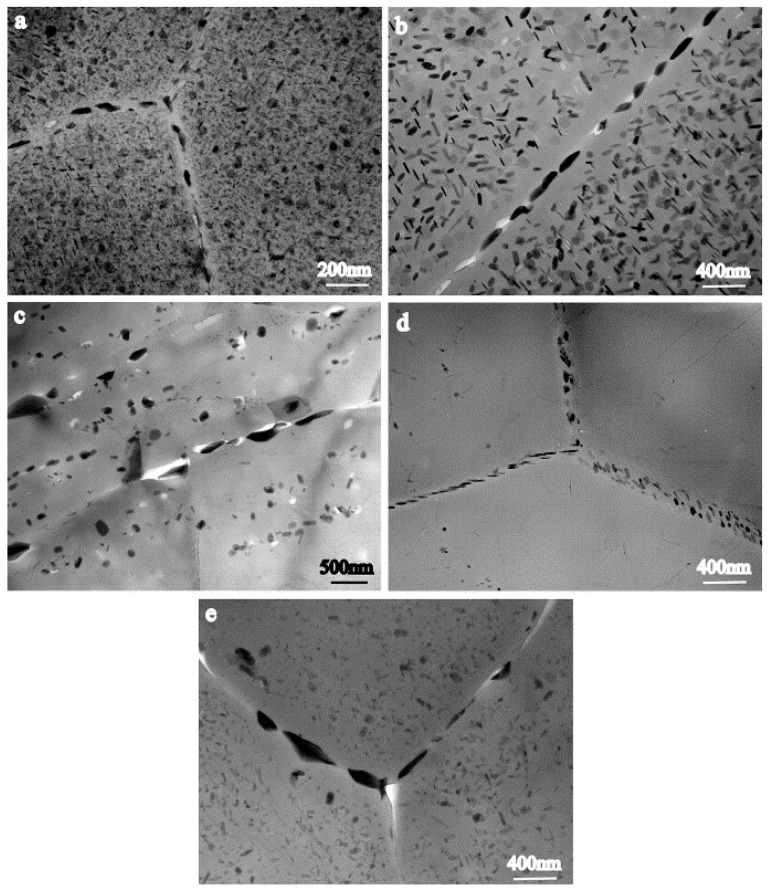
Precipitate evolution in 7050-T651 FSWeld: (**a**) base metal, (**b**) HAZ, (**c**) TMAZ I, (**d**) TMAZ II, and (**e**) SZ [43].

The TMAZ is mainly characterized by the growth and the inhibition of the η′ phase, which is conducive to the formation of equilibrium precipitates, so the hardness distribution in the TMAZ shows a downward trend [76]. In the research on FSW of 7050-T651 alloy [43], the TMAZ is divided into two parts, namely, TMAZ I near the HAZ and TMAZ II near the SZ. The peak temperature in TMAZ I is lower than that in TMAZ II. In TMAZ I, the strengthening precipitates are partially dissolved and undergo coarsening. Compared with TMAZ I, a very small amount of fine and unevenly distributed precipitates with a size of about 10–20 nm is found in TMAZ II, and precipitates are also observed at the dislocation walls (or subgrain boundaries). Since TMAZ II reaches the solution heat treatment temperature (Figure 7), the strengthening precipitates are completely dissolved. Then, during the cooling process, the dissolved precipitate phases predominantly reprecipitate along grain boundaries, subgrains and dislocation cores.

After the FSW of 7055-T6 aluminum alloy, large η precipitates were observed in the TMAZ [77]. During the FSW process, the TMAZ typically experiences the combined effects of a fairly high temperature and high shear strain. The peak temperature in the TMAZ is lower and the exposure time under high-temperature interaction is shorter than that in the SZ. Similar phase transformations may occur in the TMAZ as in the SZ: η′ phases are transformed to η by the heat effect, and η phases can survive in the TMAZ, resulting in lower concentrations of Zn and Mg solutes in the solid solution. During cooling, the η precipitates can continue to grow by absorbing Zn and Mg solutes around them and eventually becoming larger than those in the SZ. Thus, the weld hardness in the TMAZ is reduced.

#### 3.3.3. Heat-Affected Zone (HAZ)

The peak temperature in the HAZ of a 7075-T651 friction-stir-welded joint is above 250 °C [58], which exceeds the dissolution temperature of η′ precipitates (180–260 °C). The HAZ is characterized by the dissolution of fine intragranular precipitates and the growth of larger precipitates. In a study of 7050-T651 friction-stir-welded joints [43], a large amount of η′ phases were observed to remain in the HAZ; this observation suggests that exposure time above the dissolution temperature of η′ precipitates is not enough to completely dissolve the larger η′ precipitates. While the fine precipitates are dissolved into the matrix in a solid solution state, the temperature in the HAZ is not enough to dissolve the coarse phases at the grain boundary. The η phase grows by absorbing the Zn and Mg ions in the matrix, which reduces the solid solution concentration around the grain boundary. This reduces the precipitation potential of the strengthening phases and makes the dissolved strengthening phases unable to reprecipitate, causing the PFZ to become wider, as shown in Figure 7.

**Figure 8:**
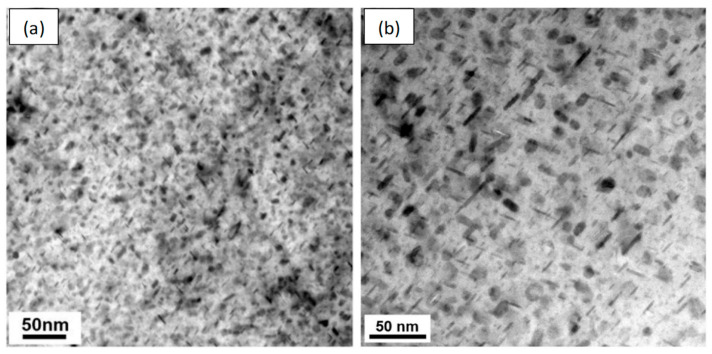
Precipitate distribution: (**a**) 7055-T6 base metal; (**b**) in HAZ of FSWeld [77].

A friction-stir-welded joint of 7055-T6 alloy was observed under a TEM. In the HAZ of the weld [77], a series of second-phase precipitates covering the Al matrix were visible; mainly η′ phases and a small amount of stable η phases were present. Most of the precipitates in the HAZ became larger than those in the base metal and partially transformed into stable η phases during FSW thermal cycling (Figure 8). Due to the coarsening and transformation of the strengthening precipitates, two softening zones formed on the advancing and retreating sides of the FSWeld, with the center as the axis. The HAZ has the characteristic of non-monotonic hardness change.

A study on the FSW of 7075-T651 aluminum alloy [78] showed that the precipitates in the HAZ were larger than those in the base metal, as determined by TEM investigation. Based on a small GP zone dissolution peak and a weak η precipitation peak on the differential scanning calorimetry (DSC) curves in the HAZ, it was believed that η′ had been dissolved and transformed into other precipitates during the FSW thermocycle, but a small amount of GP zones reprecipitated after the post-weld cooling process. The weak precipitation peak for the η phases indicates that a large amount of η appeared before the DSC test (i.e., formed after the welding heating cycle). From this combined with TEM analysis, η phases were considered to be the main precipitates in the HAZ of the 7075-T651 friction-stir-welded joint.

**Figure 9:**
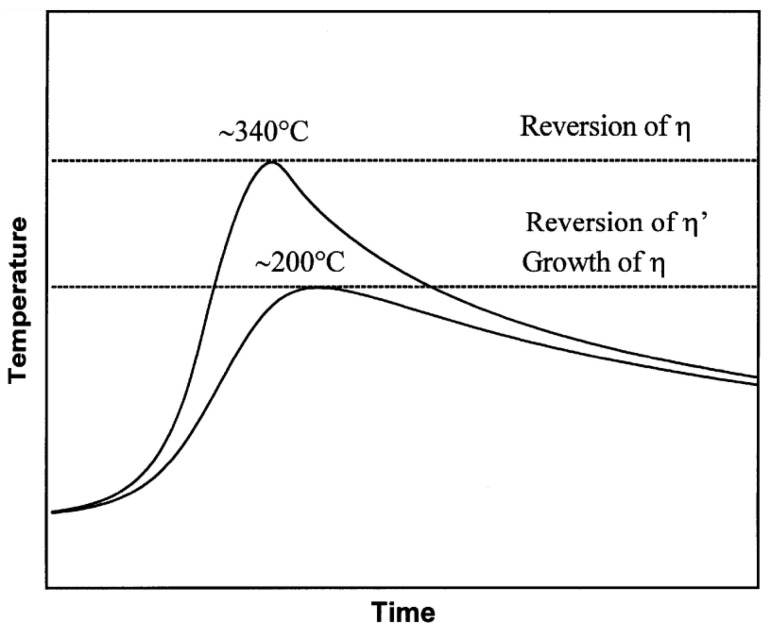
Schematic diagram of the details of the evolution of the microstructure of the HAZ during FSW of AA7108-T6 [69].

Ø. Frigaard et al. used a model that combines chemical thermodynamics and diffusion theory to describe the precipitation dissolution, reprecipitation and natural aging kinetics that occur in alloys during FSW to capture the microstructure evolution in the HAZ during the FSW of 7xxx aluminum alloys. The microstructural evolution in the HAZ of an AA7108-T651 friction-stir-welded joint is shown in Figure 9 [69]. The dissolution of the strengthening η′ phases during FSW is the main factor causing the loss of weld strength. At the same time, the growth of non-strengthening η phases depletes the solute elements in the aluminum matrix, reducing the precipitation potential; even under long-term natural aging, the strengthening phases cannot be reprecipitated, which results in the formation of a permanent soft zone in the HAZ of the weld.

## 4. Conclusions

Soluble constituent particles undergo thermal cycles during the FSW process and dissolve in the SZ and TMAZ, which has a beneficial effect on toughness. Insoluble particles are crushed and redistributed in the weld under the stirring action of the FSW process.

Dispersoids are not affected by thermal cycles or mechanical deformation during the FSW process and exist stably in the weld. Dispersoids can inhibit recrystallization and grain growth while serving as the nucleation sites of strengthening precipitates.

The fine coherent η′ precipitates are the most effective strengthening phases in Al-Zn-Mg alloys. The FSW process for aluminum alloys has a wide range of peak temperatures, and the microstructure of each weld zone is closely related to the different thermal cycles during FSW. During welding, as the tool rotates and generates heat (heating process), the thermosensitive η′ phases in the alloy may be partially dissolved, coarsening or transforming into stable η phases; then, welding reaches the peak temperature and completely dissolve under a long exposure time to high temperature. During the post-weld cooling process, the weld is naturally aged at room temperature; η′ phases may be reprecipitated or retained in a solid solution state in the Al matrix due to excessive cooling rates depending on various welding parameters.

The SZ experiences the highest peak temperature during the FSW process, which leads to the complete dissolution of the η′ strengthening phases, resulting in a hardness reduction in the SZ. The fine grain structure produced by recrystallization under plastic deformation, caused by the stirring action of the tool in the SZ, and the reprecipitation of dissolved strengthening precipitates under certain welding conditions can offset part of the hardness loss.

The TMAZ and HAZ, as transition zones, experience lower temperatures and shorter high-temperature exposure times than the SZ. In the TMAZ, fine η′ phases may be partially dissolved (and may reprecipitate after welding), and some larger η′ phases may continue to coarsen or transform into stable η phases. The η phases then continue to coarsen under heating, reducing the hardness of the material. η is considered to be the main phase in the HAZ, so a permanent softening zone forms in the HAZ.

**Figure 10:**
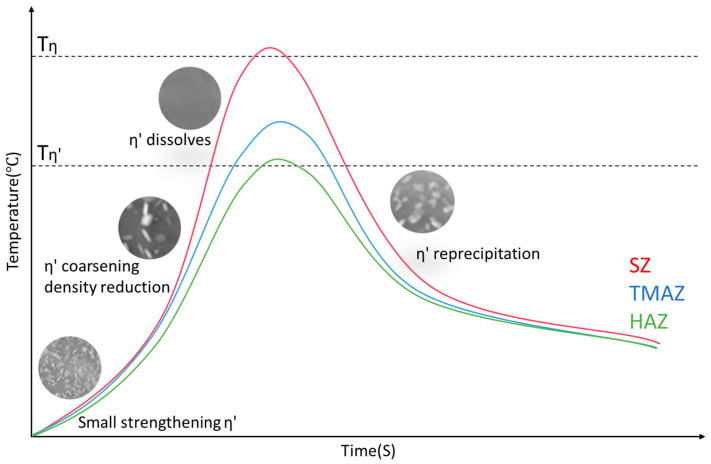
Schematic diagram of the strengthening phase evolution in different zones during FSW.

**Figure 11:**
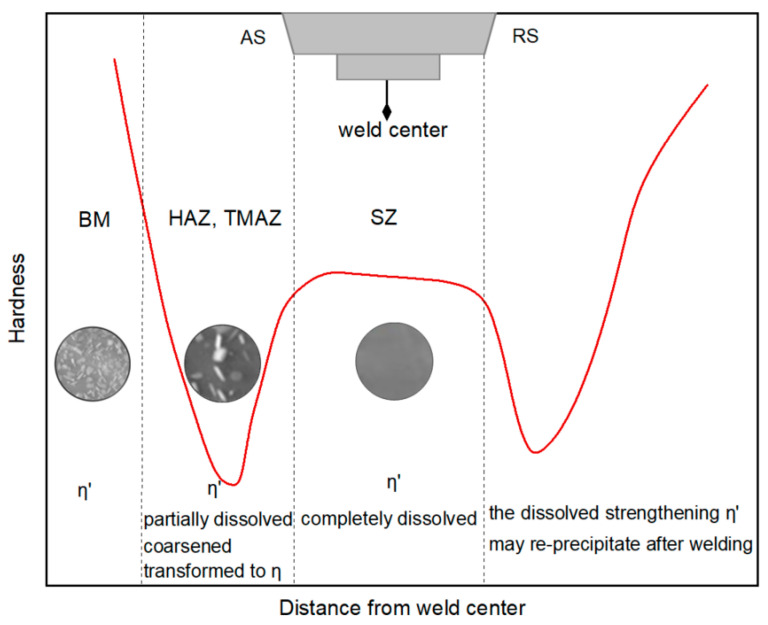
Schematic diagram of the relationship between the hardness distribution and the strengthening precipitates in a FSW joint.

The precipitate evolution behavior and the relationship between the strengthening phase evolution and uneven hardness distribution of friction-stir-welded Al-Zn-Mg alloy joints are shown in Figure 10 and Figure 11, respectively.

## References

[1] Safarbali B., Shamanian M., Eslami A. (2018). Effect of post-weld heat treatment on joint properties of dissimilar friction stir welded 2024-T4 and 7075-T6 aluminum alloys. Trans. Nonferrous Met. Soc. China.

[2] Wahid M.A., Khan Z.A., Siddiquee A.N. (2018). Review on underwater friction stir welding: A variant of friction stir welding with great potential of improving joint properties. Trans. Nonferrous Met. Soc. China.

[3] De Backer J., Bolmsjö G., Christiansson A.K. (2014). Temperature control of robotic friction stir welding using the thermoelectric effect. Int. J. Adv. Manuf. Technol..

[4] Liu G., Murr L.E., Niou C.S., McClure J.C., Vega F.R. (1997). Microstructural aspects of the friction—Stir welding of 6061-T6 aluminum. Scr. Mater..

[5] Mishra R.S., Ma Z.Y. (2005). Friction stir welding and processing. Mater. Sci. Eng. R Rep..

[6] Ahmed M.M.Z., El-Sayed Seleman M.M., Fydrych D., Çam G. (2023). Friction Stir Welding of Aluminum in the Aerospace Industry: The Current Progress and State-of-the-Art Review. Materials.

[7] Habba M.I.A., Alsaleh N.A., Badran T.E., El-Sayed Seleman M.M., Ataya S., El-Nikhaily A.E., Abdul-Latif A., Ahmed M.M.Z. (2023). Comparative Study of FSW, MIG, and TIG Welding of AA5083-H111 Based on the Evaluation of Welded Joints and Economic Aspect. Materials.

[8] Upadhyay P., Reynolds A.P. (2010). Effects of thermal boundary conditions in friction stir welded AA7050-T7 sheets. Mater. Sci. Eng..

[9] Ren S.R., Ma Z.Y., Chen L.Q. (2008). Effect of initial butt surface on tensile properties and fracture behavior of friction stir welded Al–Zn–Mg–Cu alloy. Mater. Sci. Eng. A.

[10] Fu R.D., Sun Z.Q., Sun R.C., Li Y., Liu H.J., Liu L. (2011). Improvement of weld temperature distribution and mechanical properties of 7050 aluminum alloy butt joints by submerged friction stir welding. Mater. Des..

[11] Sivaraj P., Seeman M., Seetharaman R., Balasubramanian V. (2021). Fracture toughness properties and characteristics of Friction stir welded high strength aluminium alloy by post weld heat treatment (PWHT). Mater. Today Proc..

[12] Wang S.C., Starink M.J. (2005). Precipitates and intermetallic phases in precipitation hardening Al–Cu–Mg–(Li) based alloys. Int. Mater. Rev..

[13] Dæhli L.E.B., Olufsen S.N., Kristensen T.A., Børvik T., Hopperstad O.S. (2023). Influence of constituent particles on fracture of aluminum alloys under high-triaxiality loading. Mater. Sci. Eng. A.

[14] Qian X., Parson N., Chen X.G. (2019). Effects of Mn addition and related Mn-containing dispersoids on the hot deformation behavior of 6082 aluminum alloys. Mater. Sci. Eng. A.

[15] Kosari A., Tichelaar F., Visser P., Zandbergen H., Terryn H., Mol J.M.C. (2020). Dealloying-driven local corrosion by intermetallic constituent particles and dispersoids in aerospace aluminium alloys. Corros. Sci..

[16] Fan X., Jiang D., Meng Q., Zhong L. (2006). The microstructural evolution of an Al–Zn–Mg–Cu alloy during homogenization. Mater. Lett..

[17] Que Z., Wang Y., Mendis C.L., Fang C., Xia J., Zhou X., Fan Z. (2022). Understanding Fe-Containing Intermetallic Compounds in Al Alloys: An Overview of Recent Advances from the LiME Research Hub. Metals.

[18] Prasad N., Eswara T.R., Prasad N.E., Gokhale A.A., Wanhill. R.J.H. (2014). Ramachandran. Chapter 3—Phase Diagrams and Phase Reactions in Al–Li Alloys. Aluminum—Lithium Alloys: Processing, Properties, and Applications.

[19] Zhang Y., Yang H., Sun P., Huang R., Zheng S., Duan Y., Li M. (2024). Effect of Aging Time on Precipitation of MgZn2 and Microstructure and Properties of 7075 Aluminum Alloy. J. Mater. Eng. Perform..

[20] Lynch S.P., Wanhill R.J.H., Byrnes R.T., Bray G.H., Prasad N.E., Gokhale A.A., Wanhill. R.J.H. (2014). Chapter 13—Fracture Toughness and Fracture Modes of Aerospace Aluminum–Lithium Alloys. Aluminum—Lithium Alloys: Processing, Properties, and Applications.

[21] Sharma M.M. (2008). Microstructural and mechanical characterization of various modified 7XXX series spray formed alloys. Mater. Charcterization.

[22] Starke E.A., Staley J.T., Roger L. (2011). Chapter 24—Application of modern aluminium alloys to aircraft. Fundamentals of Aluminium Metallurgy: Production, Processing and Applications.

[23] Chen S.Y., Huang C.Y., Lin C.S. (2021). Microstructure inhomogeneity of the constituent particles of 7075-T6 aluminum alloy after alkaline cleaning and desmutting. Corros. Sci..

[24] Mallinson C.F., Yates P.M., Baker M.A., Castle J.E., Harvey A., Watts J.F. (2017). The localised corrosion associated with individual second phase particles in AA7075-T6: A study by SEM, EDX, AES, SKPFM and FIB-SEM. Mater. Corros..

[25] Dorin T., Vahid A., Lamb J., Roger L. (2018). Chapter 11—Aluminium Lithium Alloys. Fundamentals of Aluminium Metallurgy: Production, Processing and Applications.

[26] Cassell A.M., Robson J.D., Race C.P., Eggeman A., Hashimoto T., Besel M. (2019). Dispersoid composition in zirconium containing Al-Zn-Mg-Cu (AA7010) aluminium alloy. Acta Mater..

[27] Liu F., Zheng J., Chen X., Xu X., Chen B. (2022). Study on corrosion resistance of artificially aged 7075 aluminium alloy by using Cs-corrected STEM. Trans. Nonferrous Met. Soc. China.

[28] Fan Y., Tang X., Wang S., Chen B. (2021). Comparisons of age hardening and precipitation behavior in 7075 alloy under single and double stage aging treatments. Met. Mater. Int..

[29] Arnoldt A., Semmelrock L., Soukup D., Österreicher J.A. (2022). Analysis of second phase particles in metals using deep learning: Segmentation of nanoscale dispersoids in 6xxx series aluminum alloys (Al-Mg-Si). Mater. Charact..

[30] Ding L., Zhao L., Weng Y., Schryvers D., Liu Q., Idrissi H. (2021). Atomic-scale investigation of the heterogeneous precipitation in the E(Al18Mg3Cr2) dispersoid of 7075 aluminum alloy. J. Alloys Compd..

[31] Dai Y., Yan L., Hao J. (2022). Review on Micro-Alloying and Preparation Method of 7xxx Series Aluminum Alloys: Progresses and Prospects. Materials.

[32] Morere B., Shahani R., Maurice C., Driver J. (2021). The influence of Al3Zr dispersoids on the recrystallization of hot-deformed AA 7010 alloys. Metall. Mater. Trans. A.

[33] Thompson D.S., Subramanya B.S., Levy S.A. (1971). Quench Rate Effects in AI-Zn-Mg-Cu Alloys. Metall. Trans..

[34] Bhuiyan M.S., Toda H., Uesugi K., Takeuchi A., Watanabe Y. (2020). Damage micromechanisms in high Mn and Zn content 7XXX aluminum alloys. Mater. Sci. Eng. A.

[35] Rometsch P.A., Zhang Y., Knight S. (2014). Heat treatment of 7xxx series aluminium alloys—Some recent developments. Trans. Od Nonferrous Met. Soc. China.

[36] Zhou B., Liu B., Zhang S. (2021). The Advancement of 7XXX Series Aluminum Alloys for Aircraft Structures: A Review. Materials.

[37] Stiller K., Warren P.J., Hansen V., Angenete J., Gjønnes J. (1999). Investigation of precipitation in an Al–Zn–Mg alloy after two-step ageing treatment at 100° and 150 °C. Mater. Sci. Eng. A.

[38] Jiang X.J., Tafto J., Noble B., Holme B., Waterloo G. (2000). Differential scanning calorimetry and electron diffraction investigation on low-temperature aging in Al-Zn-Mg alloys. Metall. Mater. Trans. A.

[39] Berg L.K., Gjønnes J., Hansen V.X., Li X.Z., Knutson-Wedel M., Schryvers D., Wallenberg L.R. (2001). GP-zones in Al–Zn–Mg alloys and their role in artificial aging. Acta Mater..

[40] Yan Z.O.U., Wu X.D., Tang S.B., Kai Z.H.A.O., Cao L.F. (2022). Tailoring phase fractions of T’ and η’ phases in dual-phase strengthened Al−Zn−Mg−Cu alloy via ageing treatment. Trans. Nonferrous Met. Soc. China.

[41] Priya P., Johnson D.R., Krane M.J. (2017). Precipitation during cooling of 7XXX aluminum alloys. Comput. Mater. Sci..

[42] Mishra R.S., Komarasamy M. (2016). Chapter 24—Physicao Metallurgy of 7XXX Alloys. Friction Stir Welding of High Strength 7XXX.

[43] Su J.Q., Nelson T.W., Mishra R., Mahoney M.J.A.M. (2003). Microstructural investigation of friction stir welded 7050-T651 aluminum. Acta Mater..

[44] Charit I., Mishra R.S. (2004). Evaluation of microstructure and superplasticity in friction stir processed 5083 Al alloy. J. Mater. Res..

[45] Gallais C., Simar A., Fabregue D., Denquin A., Lapasset G., de Meester B., Brechet Y., Pardoen T. (2007). Multiscale Analysis of the Strength and Ductility of AA 6056 Aluminum Friction Stir Welds. Metall. Mater. Trans. A.

[46] Jata K.V., Sankaran K.K., Ruschau J.J. (2000). Friction-stir welding effects on microstructure and fatigue of aluminum alloy 7050-T7451. Metall. Mater. Trans. A.

[47] Zhang J., Upadhyay P., Hovanski Y., Field D.P. (2018). High-Speed Friction Stir Welding of AA7075-T6 Sheet: Microstructure, Mechanical Properties, Micro-texture, and Thermal History. Metall. Mater. Trans. A.

[48] Feng A.H., Chen D.L., Ma Z.Y., Ma W.Y., Song R.J. (2014). Microstructure and Strain Hardening of a Friction Stir Welded High-Strength Al–Zn–Mg Alloy. Acta Metall. Sin. Engl. Lett..

[49] Wang K., Naumov A., Gushchina M., Isupov F., Alkhalaf A.A., Panchenko O. (2023). The effect of impulses on precipitation behavior in 7075-T6 aluminum alloy joint by Impulse Friction Stir Welding. Int. J. Adv. Manuf. Technol..

[50] Naumov A., Isupov F., Rylkov E., Polyakov P., Panteleev M., Skupov A., Amancio-Filho S.T., Panchenko O. (2020). Microstructural evolution and mechanical performance of Al–Cu–Li alloy joined by friction stir welding. J. Mater. Res. Technol..

[51] Farzadi A. (2017). Correlation between precipitate microstructure and mechanical properties in AA7075-T6 aluminum alloy friction stir welded joints. Mater. Sci. Eng. Technol..

[52] Gharavi F., Matori K.A., Yunus R., Othman N.K., Fadaeifard F., Fadaeifard F. (2016). Corrosion evaluation of friction stir welded lap joints of AA6061-T6 aluminum alloy. Trans. Nonferrous Met. Soc. China.

[53] Feng A.H., Chen D.L., Ma Z.Y. (2010). Microstructure and Cyclic Deformation Behavior of a Friction-Stir-Welded 7075 Al Alloy. Metall. Mater. Trans. A.

[54] Olea C.A.W., Roldo L., Dos Santos J.F., Strohaecker T.R. (2007). A sub-structural analysis of friction stir welded joints in an AA6056 Al-alloy in T4 and T6 temper conditions. Mater. Sci. Eng. A.

[55] Svensson L.E., Karlsson L., Larsson H., Karlsson B., Fazzini M., Karlsson J. (2000). Microstructure and mechanical properties of friction stir welded aluminium alloys with special reference to AA 5083 and AA 6082. Sci. Technol. Weld. Join..

[56] Morozova I., Obrosov A., Naumov A., Królicka A., Golubev I., Bokov D.O., Doynov N., Weiß S., Michailov V. (2021). Impact of Impulses on Microstructural Evolution and Mechanical Performance of Al-Mg-Si Alloy Joined by Impulse Friction Stir Welding. Materials.

[57] Simar A., Bréchet Y., de Meester B., Denquin A., Pardoen T. (2008). Microstructure, local and global mechanical properties of friction stir welds in aluminium alloy 6005A-T6. Mater. Sci. Eng. A.

[58] Mahoney M.W., Rhodes C.G., Flintoff J.G., Bingel W.H., Spurling R.A. (1998). Properties of friction-stir-welded 7075 T651 aluminum. Metall. Mater. Trans. A.

[59] Fuller C.B., Mahoney M.W., Calabrese M., Micona L. (2010). Evolution of microstructure and mechanical properties in naturally aged 7050 and 7075 Al friction stir welds. Mater. Sci. Eng. A.

[60] Sato Y.S., Kokawa H., Enomoto M., Jogan S. (1999). Microstructural Evolution of 6063 Aluminum during FrictionStir Welding. Metall. Mater. Trans. A.

[61] Dong P., Li H., Sun D., Gong W., Liu J. (2013). Effects of welding speed on the microstructure and hardness in friction stir welding joints of 6005A-T6 aluminum alloy. Mater. Des..

[62] Lee W.B., Yeon Y.M., Jung S.B. (2003). Evaluation of the microstructure and mechanical properties of friction stir welded 6005 aluminum alloy. Mater. Sci. Technol..

[63] Heinz B., Skrotzki B. (2002). Characterization of a friction-stir-welded aluminum alloy 6013. Metall. Mater. Trans. B.

[64] Hamilton C., Dymek S., Blicharski M., Brzegowy W. (2007). Microstructural and flow characteristics of friction stir welded aluminium 6061-T6 extrusions. Sci. Technol. Weld. Join..

[65] Woo W., Choo H., Brown D.W., Feng Z. (2007). Influence of the Tool Pin and Shoulder on Microstructure and Natural Aging Kinetics in a Friction-Stir-Processed 6061–T6 Aluminum Alloy. Metall. Mater. Trans. A.

[66] Dumont M., Steuwer A., Deschamps A., Peel M., Withers P.J. (2006). Microstructure mapping in friction stir welds of 7449 aluminium alloy using SAXS. Acta Mater..

[67] Zhang F., Su X., Chen Z., Nie Z. (2015). Effect of welding parameters on microstructure and mechanical properties of friction stir welded joints of a super high strength Al–Zn–Mg–Cu aluminum alloy. Mater. Des..

[68] Sullivan A., Robson J.D. (2008). Microstructural properties of friction stir welded and post-weld heat-treated 7449 aluminium alloy thick plate. Mater. Sci. Eng. A.

[69] Frigaard Ø., Grong Ø., Midling O.T. (2001). A Process Model for Friction Stir Welding of Age Hardening Aluminum Alloys. Metall. Mater. Trans. A.

[70] Bayazid S.M., Farhangi H., Asgharzadeh H., Radan L., Ghahramani A., Mirhaji A. (2016). Effect of cyclic solution treatment on microstructure and mechanical properties of friction stir welded 7075 Al alloy. Mater. Sci. Eng. A.

[71] Sivaraj P., Kanagarajan D., Balasubramanian V. (2014). Effect of post-weld heat treatment on tensile properties and microstructure characteristics of friction stir welded armour grade AA7075-T651 aluminium alloy. Def. Technol..

[72] dos Santos J.F., Staron P., Fischer T., Robson J.D., Kostka A., Colegrove P., Wang H., Hilgert J., Bergmann L., Hütsch L.L. (2018). Understanding precipitate evolution during friction stir welding of Al-Zn-Mg-Cu alloy through in-situ measurement coupled with simulation. Acta Mater..

[73] Zhao Y., Dong C., Wang C., Miao S., Tan J., Yi Y. (2020). Microstructures Evolution in Refill Friction Stir Spot Welding of Al-Zn-Mg-Cu Alloy. Materials.

[74] Jacquin D., Guillemot G. (2021). A review of microstructural changes occurring during FSW in aluminium alloys and their modelling. J. Mater. Process. Technol..

[75] Kalinenko A., Kim K., Vysotskiy I., Zuiko I., Malopheyev S., Mironov S., Kaibyshev R. (2020). Microstructure-strength relationship in friction-stir welded 6061-T6 aluminum alloy. Mater. Sci. Eng. A.

[76] Heidarzadeh A., Mironov S., Kaibyshev R., Çam G., Simar A., Gerlich A., Khodabakhshi F., Mostafaei A., Field D.P., Robson J.D. (2021). Friction stir welding/processing of metals and alloys: A comprehensive review on microstructural evolution. Prog. Mater. Sci..

[77] Lin H., Wu Y., Liu S. (2018). Impact of initial temper of base metal on microstructure and mechanical properties of friction stir welded AA 7055 alloy. Mater. Charact..

[78] Li D., Yang X., Cui L., He F., Zhang X. (2015). Investigation of stationary shoulder friction stir welding of aluminum alloy 7075-T651. J. Mater. Process. Technol..

